# Roles of the Glucocorticoid and Mineralocorticoid Receptors in Skin Pathophysiology

**DOI:** 10.3390/ijms19071906

**Published:** 2018-06-29

**Authors:** Lisa M. Sevilla, Paloma Pérez

**Affiliations:** Instituto de Biomedicina de Valencia (IBV)-CSIC, 46010 Valencia, Spain; lsevilla@ibv.csic.es

**Keywords:** glucocorticoids, glucocorticoid receptor, mineralocorticoid receptor, skin homeostasis, epidermal keratinocytes, transgenic mouse models, differentiation, inflammation, mitogen-activated protein kinase (MAPK) nuclear factor-kappaB (NF-κB) signaling

## Abstract

The nuclear hormone receptor (NR) superfamily comprises approximately 50 evolutionarily conserved proteins that play major roles in gene regulation by prototypically acting as ligand-dependent transcription factors. Besides their central role in physiology, NRs have been largely used as therapeutic drug targets in many chronic inflammatory conditions and derivatives of their specific ligands, alone or in combination, are frequently prescribed for the treatment of skin diseases. In particular, glucocorticoids (GCs) are the most commonly used compounds for treating prevalent skin diseases such as psoriasis due to their anti-proliferative and anti-inflammatory actions. However, and despite their therapeutic efficacy, the long-term use of GCs is limited because of the cutaneous adverse effects including atrophy, delayed wound healing, and increased susceptibility to stress and infections. The GC receptor (GR/*NR3C1*) and the mineralocorticoid receptor (MR/*NR3C2*) are members of the NR subclass NR3C that are highly related, both structurally and functionally. While the GR is ubiquitously expressed and is almost exclusively activated by GCs; an MR has a more restricted tissue expression pattern and can bind GCs and the mineralocorticoid aldosterone with similar high affinity. As these receptors share 95% identity in their DNA binding domains; both can recognize the same hormone response elements; theoretically resulting in transcriptional regulation of the same target genes. However, a major mechanism for specific activation of GRs and/or MRs is at the pre-receptor level by modulating the local availability of active GCs. Furthermore, the selective interactions of each receptor with spatio-temporally regulated transcription factors and co-regulators are crucial for the final transcriptional outcome. While there are abundant genome wide studies identifying GR transcriptional targets in a variety of tissue and cell types; including keratinocytes; the data for MR is more limited thus far. Our group and others have studied the role of GRs and MRs in skin development and disease by generating and characterizing mouse and cellular models with gain- and loss-of-function for each receptor. Both NRs are required for skin barrier competence during mouse development and also play a role in adult skin homeostasis. Moreover, the combined loss of epidermal GRs and MRs caused a more severe skin phenotype relative to single knock-outs (KOs) in developing skin and in acute inflammation and psoriasis, indicating that these corticosteroid receptors play cooperative roles. Understanding GR- and MR-mediated signaling in skin should contribute to deciphering their tissue-specific relative roles and ultimately help to improve GC-based therapies.

## 1. Introduction

The glucocorticoid (GC) and mineralocorticoid (MC) receptors, GR and MR, respectively, are highly homologous members of the nuclear receptor (NR) superfamily, whose roles in skin development and physiopathology have come under increasing scrutiny in recent years with the aim to improve pharmacological treatments for inflammatory skin diseases [1,2,3,4]. Both NRs can be activated by binding to GCs (cortisol, humans; corticosterone, mice) or MCs (aldosterone); however, MR binds ligand with a 10-fold higher affinity than GR [5,6,7]. Moreover, GC plasma levels are 2–3 orders of magnitude higher than those of aldosterone, meaning that without additional regulatory mechanisms, MR would be permanently occupied by GCs [5,8]. This scenario is avoided by the tissue-specific expression of the GC-inactivating enzyme 11 beta hydroxysteroid dehydrogenase type 2 (HSD11B2). Once GR and MR bind ligand, they undergo conformational changes resulting in release from cytoplasmic chaperone complexes, homo-or hetero-dimerization, nuclear translocation and regulation of target genes by binding to DNA sequences known as GC response elements (GREs) [6,7]. In addition to the similarities in these NRs, GR, and MR exhibit important differences in tissue expression patterns and biological activities [9,10,11]. Glucocorticoid receptor is ubiquitously expressed and its classical roles include regulation of cellular proliferation and differentiation, metabolism, immunity and stress response. On the other hand, the expression of MR is more limited with highest levels found in the kidney where it controls blood pressure and extracellular volume homeostasis by promoting sodium reabsorption. Glucocorticoid receptor is a well-known anti-inflammatory mediator; however, MR has been thought to be pro-inflammatory as its activation can provoke inflammation and fibrosis in cardiovascular, renal, and adipose tissues [10,12]. In contrast, recent studies have shown that in the skin MR, like GR, can also play anti-inflammatory roles [13,14].

The skin is a vital interface between the body and its surroundings, providing sensory perception and thermoregulation and protecting from dehydration, infection and mechanical stress [15]. Three principal layers compose this organ: the innermost hypodermis, which contains mainly adipocytes; the dermis, populated by fibroblasts and immune cells; and the outermost keratinized epidermis, which includes hair follicles, sebaceous and sweat glands. Importantly these distinct layers do not exist in isolation, but interact with each other via production of cytokines, growth factors, hormones, and extracellular matrix proteins [16]. The epidermis is a stratified squamous epithelium consisting mainly of keratinocytes. Stem and proliferating keratinocytes are restricted to epidermal basal layer. Upon initiation of terminal differentiation, cells migrate upwards, stop proliferating, and undergo dramatic changes in gene expression [17]. This process culminates in the formation of the stratum corneum (SC), the last line of defense from the external environment, which is composed of corneocytes, dead keratinocytes containing a highly-specialized protein and lipid matrix. Corneocytes are ultimately lost through desquamation and replaced by newly differentiated cells, a process resulting in the regeneration of this tissue every 6–8 weeks in humans and 8–10 days in mice [18]. The balance between epidermal proliferation and differentiation is tightly regulated and abnormalities can result in pathologies ranging from cancer to inflammatory skin disease.

Synthetic GCs are the most widely prescribed agent for inflammatory skin diseases including atopic dermatitis and psoriasis, affecting approximately 10–20% and 2% of populations, respectively, in developed countries [19,20]. In addition to negative effects on life quality, these diseases can progress to or be comorbid with more severe pathologies, such as asthma and rhinitis in the case of atopic dermatitis and psoriatic arthritis and cardiovascular disorders in that of psoriasis. Unfortunately, the use of GCs is limited by undesired side effects that range from osteoporosis, obesity and muscle wasting to skin thinning and impaired wound healing [21]. Deciphering the cause of these negative side effects and developing improved pharmacological agents is an ongoing area of research. In the case of GR, a great deal of effort has centered on separating its beneficial anti-inflammatory actions from the detrimental ones which impair skin integrity. Mineralocorticoid receptor, on the other hand, has been hypothesized to have exclusively negative actions when activated by pharmacological doses of GCs due to the pathological effects it exerts in other tissues. While a great deal of interest has been placed on the pharmacology of these receptors, until recently much less was known about their roles in skin development and homeostasis.

## 2. Transcriptional Regulation by GR and MR

Glucocorticoid receptors and MRs are descended from an ancestral corticoid receptor [22] and share the primary domain structure of the NR superfamily, consisting of an N-terminal transactivation domain, a central DNA-binding domain and a C-terminal ligand-binding domain [23,24]. The N-terminal domain interacts with the transcriptional machinery and co-regulators via the Activation Function (AF)-1 region in GR and the AF-1a, middle domain and AF-1b in MR [23,24]. The MR N-terminus also contains a ligand-independent nuclear localization signal [25]. The majority of posttranslational modification sites, including those for phosphorylation, ubiquitination and sumoylation, are present in the N-terminal domains of GR and MR, allowing for modulation of receptor function and contributing to context specificity [23,25]. The DNA-binding domains of these receptors are highly conserved with two zinc finger motifs that recognize and bind GREs as well as a nuclear localization signal. In both GR and MR, the DNA-binding domain is connected via a flexible hinge region to the C-terminal ligand-binding domain that contains a hydrophobic pocket for ligand binding, an AF-2 region for ligand-dependent interactions with co-regulators and an additional nuclear localization signal. 

*NR3C1*, the gene encoding GR, contains nine exons and its transcript can undergo alternative splicing resulting in at least five isoforms [6], with GRα and GRβ differing at their C-termini, being the most studied to date. While GRα binds ligand and mediates classical receptor actions GRβ cannot bind GCs and is as a dominant–negative inhibitor of GRα. GRβ is usually expressed at lower levels compared to GRα, and changes in their ratio affect GC sensitivity and are associated with autoimmune disease [6]. The gene encoding MR, *NR3C2*, consists of 10 exons and also generates multiple transcriptional isoforms [26]. One such isoform lacks the entire hinge region and the ligand-binding domain and has been shown to bind DNA and potentiate the transcriptional activation of full length MR and GR through recruitment of co-activators [27]. The scenario is even more complicated because multiple GR and MR protein isoforms are generated through alternative translational initiation [6,26]. While the MR translational isoforms have not been extensively studied, those of GR show differential tissue expression and though all are capable of binding DNA and ligand, the N-terminal truncations alter subcellular localization and transcriptional activity [6]. The precise role of MR- and GR-isoforms in skin remains to be determined.

In the absence of ligand, GRs and MRs are mainly found in the cytoplasm in a multiprotein complex that includes chaperones (heat shock protein (HSP)70, HSP90 and p23) and immunophilins [6,7,12,24]. Upon ligand binding the receptors dissociate from this complex, translocate to the nucleus, and bind to GREs or to other TFs, eliciting transcriptional responses. For GR, transcriptional regulation through direct chromatin binding requiring receptor dimerization (termed transactivation) was long thought to be responsible for the adverse effects of GC treatments. On the other hand, the positive anti-inflammatory actions of GCs were thought to occur via interference of GR with other TFs, in particular nuclear factor-kappaB (NF-κB and activator protein 1 (AP-1), independently of DNA-binding and receptor dimerization via a tethering mechanism (known as transrepression) [28,29]. This was based on studies using a single amino acid mutation (A458T) in the GR DNA-binding domain, which was reported to block dimerization and impair transactivation of GRE-containing target genes while permitting transrepression of AP-1 and NF-κB [30]. However, co-immunoprecipitation experiments identifying interactions between GR^A458T^ mutants raised questions about this model. The Numbers and Brightness microscopic technique confirmed that GR^A458T^ was capable of dimerization in live cells and chromatin immunoprecipitation experiments showed it could bind DNA though with reduced efficiency in a subset of GREs [31]. This together with results with other GR mutants that did not find a correlation between oligomeric state and transcriptional activity, suggest another explanation for the reduced transcriptional activity of GR^A458T^, such as conformational alterations affecting binding of the transcriptional machinery. The latest challenge to the dogma of GR function is in vivo evidence that GRE binding involves GR tetramers rather than dimers [32].

While over 20 chromatin immunoprecipitation (ChIP)-Seq studies have been published for GR, few have been reported for MRs [33,34,35,36]. These studies showed that GR- and MR-bound genomic regions are widespread throughout the genome, and not necessarily located in close proximity to target genes [35,37,38]. The canonical GRE sequence consists of an imperfect palindrome containing two half sites and a three base pair spacer (5′-AGA ACA nnn TGT TCT-3′). However, endogenous GREs show a great deal of variability, with changes in the majority of positions not impeding GR binding [37,39]. GR can also regulate transcription by binding to half sites [40], such as those found near the epidermal keratin (*Krt*) 5, 14, 6 and 17 genes [41]. It has been long assumed that gene induction resulted from GR binding to GREs while gene repression occurred upon GR binding to so-called negative GREs or tethering towards NF-κB or AP-1. However, genome-wide profiling in lipopolysacharide-activated macrophages demonstrated that both positive and negative GR cistromes are mainly composed of classical GREs located close to NF-κB and AP-1 binding sites [42]. This implies that cistromic motif classification is not enough to predict the polarity of the transcriptional response. These studies also showed that the GR-dependent repression of inflammatory genes involves recruitment of the NR corepressor GRIP1, leading to Interferon regulatory factor 3 antagonism and histone deacetylase (HDAC) recruitment [42]. Finally, a recently identified GC regulatory sequence is the inverted repeat (IR)-negative (n) GRE, which can be bound by GR but not MR, and has the consensus sequence 5′-CTCC (n)0-2 GGAGA-3′ [43]. These sites promote the assembly of monomeric GR and cis-acting corepressor complexes that recruit HDACs to repress gene expression. Interestingly, many GC-repressed genes in epidermal keratinocytes, including cytokines, contained IR nGRES [43].

Less than 10% of MR-binding sites (MBS) found in human renal cells contained canonical GREs and the majority of identified GREs were half sites [35]. Interestingly, most MBS (75%) identified lacked MREs altogether and instead contained sites for other transcription factors (TFs) including FOX and AP1, suggesting that MR may interact with these genomic regions indirectly through protein-protein interactions or tethering [35]. Thus far only one GR ChIP-Seq experiment has been performed in keratinocytes and in contrast to the MR ChIP-seq in human renal cells over 60% of GR-binding sites (GBS) contained GREs [35,44]. In keratinocytes, AP-1 and KLF motifs were overrepresented in the GBS, some of which were adjacent to GRE sequences, as in the case of *Tristetraprolin*/*Zfp3*6 and *Gilz*/*Tsc22d3*, which were found to be regulated by cooperation between GR and KLF4.

Glucocorticoid receptors and MRs bind to GREs as homo- or hetero-dimers or higher order complexes [32,45,46]. Sequences in the DNA- and ligand-binding domains are involved in GR homodimerization [23,31], while those in the highly conserved DNA-binding domain control that of MR [7]. In vitro studies showed that distinct homo- and hetero-dimeric combinations have different transcriptional efficiencies depending on the GRE [7]. However, until recently the functional relevance of GR-MR heterodimers in vivo was not known. In the hippocampus of stressed rats, tandem ChIP experiments indicated that GR and MR bound as homo- and hetero-dimers to *Fkbp51*, but predominantly as homodimers to other genes, suggesting differential regulatory mechanisms [47,48]. Similarly, a recent report has shown that ligand-bound GRs and MRs are recruited as homo- and hetero-dimers in response to aldosterone and cortisol to *PER1* in renal cells [49]. These experiments demonstrate that the interaction of both receptors at the same target promoter, which occurs with different kinetics, results in specific and distinct transcriptional signatures and highlights the complexity of gene regulation by GR and MR [49]. In keratinocytes, GRs and MRs can heterodimerize in response to Dex and synergistically activate a GRE-luciferase reporter [50], however the relative binding of homo- and hetero-dimers to target genes in this cell type is unknown. An area of future interest is the identification of MBS in keratinocytes, as comparison of genomic bound regions in ChIP-seq experiments carried out in different cell types is limited by the well-known context and cell-type specific actions of these TFs.

Glucocorticoid receptors and MRs are also responsible for rapid non-genomic actions that occur within seconds to minutes following ligand binding and do not require transcription or translation [21,51]. For example, ligand-bound GRs interfere with phosphatidylinositol-3-kinase signaling and the downstream kinase acutely transforming retrovirus (AKT), important for cell proliferation and survival. This interference occurs in keratinocytes and contributes to the antitumor effects of GCs in mouse skin [52]. While the non-genomic actions of MRs in keratinocytes remain to be determined, in other cell types aldosterone-MR can activate the epidermal growth factor receptor (EGFR) leading to downstream mitogen-activated protein kinase (MAPK)and/or phosphatidylinositol-3-kinase signaling [51].

## 3. Systemic and Cutaneous Glucocorticoid/Mineralocorticoid Production and Regulation

Glucocorticoids are synthesized and released in the adrenal cortex upon hypothalamic production of corticotropin-releasing hormone receptor (CRH) and release of adrenocorticotropic hormone (ACTH) from the anterior pituitary, the so-called hypothalamic-pituitary-adrenal (HPA) axis, upon pathophysiological conditions. Glucocorticoid production is limited through negative feedback via GR which inhibits the secretion of CRH and ACTH [5]. Consistent with this, GR^−/−^ mice exhibited increased plasma levels of both ACTH (>20-fold) and GCs (2- to 3-fold) relative to controls. Also, and since the expression of the steroid biosynthetic enzyme aldosterone synthase is under control of ACTH in the adrenal zona glomerulosa, the elevated levels of ACTH in GR^−/−^ mice resulted in increased plasma aldosterone levels [53]. MR^−/−^ mice also showed a strong increase in the circulating levels of aldosterone (65-fold) [54].

Local production of GCs has been found in a wide variety of cell types and tissues, including lymphoid, brain, gut, and the skin. All components of the HPA axis and steroidogenic enzymes are present and functional in the skin [55,56]. As this tissue is constantly affected by external perturbations, the local synthesis of GCs, such as that provoked by ultraviolet (UV) exposure, serves as a rapid, local response to stress [55,57]. Recent studies have emphasized the importance of local GC biosynthesis in maintaining homeostasis [58,59].

The balance between HSD11B1 and HSD11B2 activities maintains appropriate GC levels and constitutes a key mechanism to modulate NR function at the pre-receptor level [8]. HSD11B1 is expressed in human and mouse epidermis and dermis, and is upregulated in differentiating keratinocytes [60,61]. HSD11B2 is also present in human and mouse suprabasal epidermis as well as in sweat glands, an important target for aldosterone-MR regulation [10,50,62,63,64]. However, there is no evidence of local aldosterone synthesis in the epidermal or dermal cells of the skin [57]. Therefore, with the exception of pathological situations with elevated plasma aldosterone levels such as primary hyperaldosteronism or Conn’s syndrome, it is likely that the main signaling through MR in the skin is via GCs.

## 4. GC Signaling Exerts Crucial Roles in Skin Development

### 4.1. Development of the Epidermis and Its Appendages 

Developing embryos receive maternal GCs in addition to those produced systemically starting at embryonic day (E)14 and peaking around birth [53]. It is not known whether local GCs are synthesized during skin development. Epidermal differentiation and barrier formation is tightly regulated with defects leading to inflammatory skin disease later in life [65]. This process initiates at embryonic day (E)10.5 when surface ectoderm cells begin to express the keratinocyte-specific intermediate filament proteins keratins 5 and 14 that continue to be expressed in the basal layer of developing and adult epidermis [66]. Following epidermal stratification at E14.5, keratinocytes upregulate the suprabasal keratins 1 and 10 and undergo terminal differentiation to form the post-mitotic spinous and granular layers and the outermost SC [15]. The epidermal permeability barrier becomes functional around E17.5 upon maturation of the SC, which is composed of corneocytes, fully differentiated dead keratinocytes, surrounded by specialized extracellular lipids. SC maturation occurs in a time-window of approximately 24 h (E16.5–E17.5), and permeability barrier acquisition is patterned and can be visualized by whole mount dye exclusion assays [67]. Importantly, epidermal expression of both GR and MR peaks at E16.5 [57,68]. At this same timepoint, *Hsd11b1* and *Hsd11b2* are low compared to the end of gestation (E18.5) when their levels are higher by more than 10- and 30-fold, respectively [57]. Taken together, during epidermal barrier formation there are relatively high levels of GR and MR and most likely abundant active GCs. The peak of *Nr3c2* expression during development is very pronounced, with a 5-fold decrease between E18.5 and 8-week old skin [57]. Levels of *Nr3c1* on the other hand do not show such a dramatic contrast between developing and adult skin (our unpublished data).

No skin phenotype has been reported for newborn aldosterone synthase KO mice, supporting that GCs and not MCs principally regulate the development of this tissue [69]. Consistent with this, antenatal exposure to pharmacological doses of GCs accelerates permeability barrier formation [67,70], while deficiency in systemic GC synthesis impairs this process [71]. Gene expression studies indicated that a critical time window for transcriptional responsiveness of embryonic skin to maternal pharmacological GC-treatment is from E15.5 to E16.5 [72]. During this time period, GC treatment induced members of the epidermal differentiation complex, found on chromosome 3q in mice and 1q21 in humans, which comprises over 55 genes that participate in keratinocyte terminal differentiation.

### 4.2. Gain and Loss of Function Mouse Models for Studying GR and MR in Skin Development

Gain-of-function was assessed using transgenic mice expressing GRs or MRs under the control of the keratin 5 (K5) promoter that is active in all stratified epithelia, including the basal layer of the epidermis, hair follicles and sebaceous glands [73,74,75]. There were striking similarities between the K5-MR and K5-GR transgenic mice including defective skin development, an eye-open-at birth phenotype, abnormal hair follicle morphogenesis and perinatal lethality [74,75,76]. Both mouse models showed epidermal hypoplasia consistent with the anti-proliferative effects of pharmacological GC treatments on keratinocytes both in vivo and in vitro [77,78]. However, while decreased proliferation as assessed by BrdU incorporation was a consistent feature of the K5-GR, no differences in the proliferation marker Ki67 were observed in developing K5-MR epidermis [74,75]. Instead there was a dramatic increase in TUNEL positive subrabasal nuclei indicating that an increase in apoptotic keratinocytes contributed to the epidermal hypoplasia of the K5-MR. Accelerated embryonic epidermal differentiation was observed in both transgenic models (increases in loricrin staining in K5-GR and earlier dye exclusion in K5-MR) which is in line with the ability of GCs to induce keratinocyte differentiation (our unpublished data; [75,79]). Finally, an important difference between these transgenics is that the K5-GR, but not the K5-MR, showed defective development of other ectodermal derivatives including the teeth and exocrine glands, sharing phenotypic similarities with the human disease hypohidrotic ectodermal dysplasia [75,76]. The molecular bases for the observed defects are related to the impaired expression and/or activity of NF-κB and p63 in epithelia of K5-GR transgenics. Importantly, mutations in multiple components of the NF-κB signaling pathway as well as in p63, crucial regulators of epidermal function, are mutated in patients with ectodermal dysplasias. Taken together these data suggest functional interactions between GR and these TFs [18,76]. These results advise caution regarding the use of topical GCs to treat the associated dermatitis in patients with ectodermal dysplasia.

The complete loss of function GR^−/−^ mice have a strong skin phenotype that was complementary to that observed in the K5-GR model, whereas the MR^−/−^ had a far milder phenotype indicating important differences in physiological functions of GR and MR during development of this tissue [57,80]. Both KOs die postnatally, the GR^−/−^ upon birth due to defects in lung maturation and the MR^−/−^ around P10 due to renal failure, hypertension and dehydration [53,64]. The MR^−/−^ mice can be rescued from perinatal lethality by administration of NaCl and interestingly, perinatal treatment with synthetic GCs also prolonged survival indicating that in certain circumstances GR can partially substitute for MR [81,82]. Histology of developing E16.5–E18.5 GR^−/−^ skin showed striking epidermal abnormalities and dye exclusion assays confirmed that permeability barrier formation was delayed relative to control littermates [80]. Though keratinocytes underwent stratification neither the granular layer nor the SC was evident and levels of SC proteins filaggrin, loricrin, and involucrin were almost negligible [80]. In contrast, permeability barrier acquisition was normal in the MR^−/−^ and all epidermal layers were present [57]. However, at E17.5, the MR^−/−^ epidermis showed hyperplasia and had abnormal expression of K5, K10 and loricrin [57]. These minor defects resolved and upon birth MR^−/−^ skin appeared completely normal, leading to the conclusion that this NR plays a transient role during skin development coinciding with its peak of expression at E16.5. This contrasts with GR which plays a more persistent role throughout development of this tissue. Microarray analysis of E18.5 GR^−/−^ skin identified dysregulation of genes associated with ectoderm/epidermis development, including epidermal differentiation complex members such as the Small proline repeat rich (*Sprr*) family and *corneodesmosin* [68]. GR^−/−^ epidermis also exhibited abnormal suprabasal keratinocyte proliferation and increased apoptosis. An increase in phosphorylated extracellular signal regulated kinase (ERK) was observed in GR^−/−^ keratinocytes in vivo as well as in vitro where it was shown to contribute to the increased apoptosis [80].

As knock-in mouse model (GR^A458T/A458T^) was viable and fertile, unlike GR^−/−^ mice, this led to the conclusion that the transactivation function is not required for survival [53,64]. Similar conclusions were made in skin as that of newborn GR^A458T/A458T^mice had normal histological appearance and differentiation marker expression [80]. However, given that GR^A458T^ can dimerize, bind DNA, and at least partially activate a subset of target genes, the lack of skin phenotype in newborn GR^A458T/A458T^ mice does not conclusively resolve the importance of GR dimerization-mediated transactivation in this process.

To understand the effects of loss of MR and/or GR in the epidermis beyond birth, knockouts were generated using NR^flox/flox^ mice, which have the third exon, encoding part of the DNA binding domain, flanked by loxP sites (reviewed in Reference [83]). When crossed with transgenic mice that express the Cre recombinase, exon 3 is disrupted [64,84]. Developing and newborn epidermal GR KO (K5-Cre//GR^flox/flox^ or GR^EKO^) and MR KO (K5-Cre//MR^flox/flox^ or MR^EKO^) mice had different skin phenotypes, with the histological appearance of GR^EKO^ skin showing pronounced abnormalities while that of MR^EKO^ was almost identical to control littermates [13,14]. The defects in GR^EKO^ developing and newborn skin included delayed epidermal barrier formation, abnormal differentiation, increased proliferation and SC fragility [13]. Consistent with this, GR^EKO^ epidermis had decreased levels of SC proteins loricrin, filaggrin and corneodesmosin; abnormal interfollicular keratin 6 expression and alterations in epidermal lipids, all of which were normal in newborn MR^EKO^. In addition to the epidermal defects, GR^EKO^ mice had dermal infiltrates containing macrophages and degranulated mast cells, features of inflammatory skin disease. 

To determine whether these NRs cooperate or compensate for each other’s loss in the single KO mice, double GR/MR epidermal KO (DKO) mice were generated [50]. Newborn DKO mice showed a striking skin phenotype that was more severe than either single KO with regions in the epidermis where keratinocytes were unorganized and failed to flatten suprabasally, and the granular layer and SC were not well differentiated [50]. These mice also had an inflammatory phenotype with neutrophil containing epidermal microabcesses, something that was not observed in GR^EKO^ or MR^EKO^ [50]. All genotypes, even MR^EKO^ skin with its apparently normal histology, showed similar increases in epidermal phosphorylated-ERK and p38 MAPK, indicative that both GR and MR are required to antagonize inflammatory signaling pathways in this tissue [50]. Comparative analysis in newborn epidermis identified genes whose expression depended entirely on the presence of GR (*Krt77*, *Mmp3*, *Slpi*, *Tslp*) or MR (*Hsd11b2*). On the other hand, the gene *Sprr2d* required the presence of GR and MR as loss of either or both resulted in similar dysregulation. Importantly, other genes (*Lcn2*, *S100a8*) showed evidence of cooperative regulation by GR and MR as there were additive differences in expression in single KO versus DKO epidermis [50]. Through a yet unknown mechanism, the skin phenotype of GR^EKO^ and DKO resolves around postnatal day 5.

## 5. GR and MR in Adult Skin Homeostasis

### 5.1. Expression and Function of GR and MR in Cultured Keratinocytes

While both GRs and MRs are present in all skin compartments, there are differences in expression depending on cell type or stage of differentiation. For instance, GR levels show a slight increase during keratinocyte terminal differentiation, while MR is higher in undifferentiated keratinocytes [44,57,85], suggestive of differing roles in this process. Indeed, immortalized adult mouse keratinocytes lacking expression of GR (GR^EKO^) have a phenotype of decreased differentiation, while those lacking MR (MR^EKO^) show increases in this process [13,14]. DKO immortalized keratinocytes exhibited both similar and unique features relative to single KOs [50]. For example, single and double KO cell lines showed similar increases in basal p38 and ERK MAPK activities. Reinsertion of GR or MR in DKO keratinocytes reduced p38 and ERK activities to levels of control cells, however, co-transfection of both receptors did not result in further decreases. On the other hand, DKO keratinocytes displayed increased basal AP-1 and NF-κB transcriptional activities relative to single KOs, which could be partially rescued by ERK and p38 inhibition, respectively. These data suggest that while ERK and p38 activities are similarly regulated by GR or MR, these receptors exert additive or synergistic regulation of the AP-1 and NF-κB pathways through yet uncharacterized mechanisms [50]. A striking feature of single and double KO keratinocytes was their decreased size relative to control cells, with DKO cells being the smallest; suggesting interaction of GR and MR with pathways controlling cell size [50]. GR^EKO^ but not MR^EKO^ or DKO keratinocytes exhibited an epithelial-mesenchymal transition phenotype with loss of E-cadherin and up-regulation of smooth muscle actin, suggesting that GR deficiency does not trigger these alterations but they are rather due to a pathological role of MR in the absence of GR [50,86].

In vitro data have demonstrated physical and functional interaction between GR and MR in keratinocytes. When DKO cells were co-transfected with GR and MR (1:1 ratio) both NRs co-localized and physically interacted in the cytoplasm even in the absence of hormone. Upon Dex treatment this interaction was detected only in the nucleus [50]. As expected, DKO cells treated with the synthetic GC dexamethasone did not transactivate a GRE-luciferase reporter. Transfection of GR or MR restored the transcriptional response in a dose-dependent manner; however much higher doses of MR were required for reporter transactivation. Co-transfection of suboptimal levels of GR and MR, individually not capable of transactivation, showed additive effects inducing the GRE-luciferase reporter to levels seen in control cells [50]. Altogether, these data support the functional and cooperative role of these corticosteroid receptors in mediating GC transcriptional responses in keratinocytes.

### 5.2. Cutaneous Manifestations of Imbalances in GCs and Their Receptors

Glucocorticoid levels must be tightly controlled as imbalances in circulating hormone levels exert pleiotropic adverse effects. The homeostatic control exerted by the HPA axis fits with an inverted U-shaped dose-response curve, in which optimal effects are achieved in the central range of the curve while suboptimal effects (including both GC excess or deficiency) lie on either side of the curve [5,87]. In skin, GC overproduction in Cushing’s patients causes cutaneous abnormalities that include skin atrophy, impaired collagen formation, purple abdominal striae, and steroid acne. Importantly, these patients may also exhibit increased skin fragility, high risk of infection, and delayed wound healing. These alterations are very similar to those found in aging and after prolonged or high dose GC pharmacological treatments [55,88]. Also, aldosterone excess in patients with primary hyperaldosteronism may cause skin alterations that include epidermal hyperplasia, impaired differentiation, and inflammatory features associated with abnormal GR/MR activation in keratinocytes [58]. On the other hand, Addison’s patients—with deficiency in GC and MC production—show generalized hyperpigmentation as result of the elevated levels of melanocyte-stimulating hormone due to the loss of negative feedback of the HPA [89].

### 5.3. Strategies to Separate the Therapeutic and Adverse GC Effects

A major goal of in the field has been to separate the undesirable side effects from the therapeutic benefits of GC treatment, which until more recently have focused mainly on GR [28,29,90]. The classical model was that the beneficial anti-inflammatory actions of GR were due to transrepression while transactivation mediated the negative side effects of GCs [90]. A great investment of pharmacological research centered on generating agents that could dissociate the transrepression/transactivation functions of GR based on the classical monomer/dimer model. This led to development of selective GR agonists (steroidal scaffolds) or modulators (non-steroidal scaffolds) [91,92]. Compound A (CpdA), an analog of a naturally occurring compound in Salsola tuberculatiformis, was one of the first selective GR modulators to be characterized and has been extensively researched [92,93]. Treatment of cell lines with CpdA resulted in the inhibition of NF-κB and AP-1 signaling but not in the induction of classical GR target genes, thus showing the desired dissociated activity. In rodents, topical CpdA treatment reduced phorbol 12-myristate 13-acetate (PMA)-induced hyperplasia and inflammatory cytokines expression without causing skin atrophy [92,93]. There are issues with specificity of this agent, as CpdA treatment results in changes in expression of a unique gene subset, not associated with GCs; also, the high degree of chemical instability of CpdA is a barrier to its clinical use [92,93].

In addition to the mechanistic breakthroughs discussed above, it has become clear that GR transactivation plays an important beneficial role by inducing the transcription of key anti-inflammatory mediators including *Gilz*, *Zfp36*, and *Mkp1*/*Dusp1* (mitogen activated protein kinase phosphatase 1) [94]. In particular, many efforts have focused on the use of GILZ as a more specific downstream mediator of GC action, which would theoretically lack undesired side-effects [90]. However, there are apparently contradictory results regarding the role of this GC target in skin inflammation as *Gilz*^−/−^ mice and transgenic mice with generalized overexpression of GILZ were more susceptible to imiquimod-induced psoriasis [95,96].

Given the close structural and functional homology of GR and MR, as well as the similarities in the skin phenotypes of K5-GR and K5-MR or adult GR^EKO^ and MR^EKO^ mice (see below), we and others postulated that the effects of pharmacological GCs were mediated by both receptors [3,4,97]. The initial hypothesis was that MR was responsible for some of the undesired side-effects of excess GCs. This held true in the case of GC-induced skin atrophy as demonstrated by human and mouse models. Topical application of a pharmacological MR antagonist partially reversed GC-induced skin atrophy in healthy human skin [98]. Also, MR^EKO^ mice were partially protected against GC-induced skin atrophy with improvement in adverse effects of topical Dex (relative to wild-typecontrols) including epidermal thinning, reduced K5 expression, decreased keratinocyte proliferation, and repression of Ccnd1 [14]. These data, together with the fact that GR expression was unchanged in MR^EKO^ skin, show that MR participates in the repression of genes associated with proliferation which are known GR-targets such as *Krt5* and *Ccnd1* [14]. These findings opened the attractive possibility of using combined therapy with GCs and pharmacological MR antagonists to improve GC-induced skin atrophy. However, in a model of contact dermatitis with human skin explants, MR blockade reduced GC-induced skin atrophy, but limited the repression of some inflammatory cytokines, suggesting that MR may be required for the full therapeutic response to GCs in this tissue [99]. This possibility is supported by the observations from our laboratory that mice lacking epidermal MR, similar to those lacking GR in this tissue, are more susceptible to skin inflammation [13,14]. 

#### 5.3.1. Skin Atrophy, Delayed Wound Healing, and Aging

Skin atrophy and delayed wound healing, in addition to being major adverse effects associated with pharmacological GC treatments, are characteristics of aging skin. Skin atrophy is readily detected as the tissue becomes thinner, shiny and telagientactic and more susceptible to mechanical and environmental aggressors [77,88,100]. The histopathological features of atrophic skin include reduced thickness of both the epidermal and dermal compartments and altered deposition of extracellular matrix proteins that normally provide the support and resilience to this tissue. In adulthood, only K5-GR mice showed epidermal hypoplasia while K5-MR did not exhibit alterations in the interfollicular epidermis. However, both mouse models had alterations in hair follicles with a 50% decrease in number along with atropy of the remaining follicles in K5-GR and progressive alopecia with formation of hair follicle cysts in K5-MR mice [74,75,76]. On the other hand, loss of GR and/or MR in the epidermis did not affect hair follicle number or cycling ([13,14], and unpublished observations). However, a slight thickening of the interfollicular epidermis was observed in single and double KO adult mice relative to control animals [13,14,50]. 

After wounding, skin homeostasis is restored by the coordinated interplay of keratinocytes, immune cells and fibroblasts through a process that includes inflammation, proliferation, and remodeling. It is well known that GC imbalances (systemic or local) alter cutaneous wound healing [79,101,102,103,104,105]. Endogenous GC excess results in delayed healing and in the most severe cases, as in diabetic patients, causes non-healing chronic wounds. This delay is due to the excessive anti-proliferative and anti-inflammatory actions of GCs, which reduce the expression of growth factors and cytokines necessary for keratinocyte proliferation and migration (also called re-epithelialization) interfering with normal skin barrier repair [101,102]. 

Similar to systemic GC treatments, K5-GR mice showed delayed skin wound healing indicating that epidermal-specific GR overexpression is sufficient to interfere with this process [106]. This delayed wounding was a consequence of a decreased inflammatory response (diminished *Tnfa*, *Il1β*, *Tgfβ1* and *Kgf* expression and *Tgfβ3* up-regulation) with reduced recruitment of granulocytes and macrophages, and impaired keratinocyte migration in vitro and in vivo [106]. Importantly, K5-GR-TR mice, with keratinocyte-targeted overexpression of a mutant GR (P493R, A494S) with impaired transcriptional activation but normal transrepression function exhibited less pronounced delays in healing relative to K5-GR mice due to decreased repression of pro-inflammatory cytokines and growth factors [106,107]. Delayed wound responses in the transgenic vs control keratinocytes correlated with reduced ERK activity both in vivo and in vitro [106]. Overall, while the transrepression function of GR is sufficient for negative interference of the early stages of wound closure the GR-dependent transcriptional activation is necessary to delay the later stages of this process.

The mechanisms by which GC-activated GR inhibit wound closure involve, among others, the repression of EGF-induced keratinocyte migration by forming a repressor complex together with β-catenin that inhibits the expression of the keratins K6 and K16 at the wound edge [108]. In agreement with this, K5-GR and K5-GR-TR mice showed nuclear localization of β-catenin in keratinocytes upon wounding [106]. Other GR agonists such as farnesyl pyrophosphate, an intermediate in the cholesterol biosynthetic pathway, can also inhibit keratinocyte migration by inducing nuclear translocation of β-catenin and expression of c-myc, a feature of non-healing wounds [109]. In addition, GCs can inhibit keratinocyte migration and wound healing through non-genomic pathways that involve membrane GR and regulation of phospholipase C/protein kinase C ultimately leading to activation of β-catenin and c-myc [110,111].

Recent data have shown apparently contradictory results regarding the role of MR in cutaneous wound healing. On one hand, it was shown that MR blockade improved wound re-epithelialization in GC-treated human skin explants and in GC-treated skin of healthy volunteers in a clinical trial [112], while having no effect in skin unexposed to pharmacological GCs [98]. This was also the case with MR-blockade improving wound healing in mice with streptozotocin-induced diabetes, but not healthy controls [112]. It was postulated that the delays in healing after topical GC treatment or in the skin of diabetic mice were due to increased MR expression and activation (as the mRNA levels of the MR target *ENacα* increased). Consistent with this, skin explants from newborn K5-MR mice showed impaired epidermal outgrowth relative to controls, and the addition of an MR antagonist partially rescued the delay in healing [112]. However, assessment of wound healing in MR^EKO^ adult mice showed defects in dermal remodeling and no changes in re-epithelialization relative to controls. Wound scratch assays in cultured keratinocytes demonstrated retarded MR^EKO^ cell migration relative to controls, and consistently moreover, treatment with the MR antagonist eplerenone resulted in significant delays in keratinocyte migration at early timepoints following wounding [14]. These differences are likely due to the experimental settings, topical treatments with pharmacological MR blockers in whole skin vs epidermal-specific deletion of MR, and indicate roles for MR in other non-keratinocyte cell types during the wound healing process.

It is well known that aged skin features histopathological changes including epidermal thinning, reduced dermal volume and content of extracellular matrix proteins, all of which contribute to a defective skin barrier with reduced resistance to external insults. The reported increased expression of HSD11B1 in skin during aging—despite the lack of changes in plasma cortisol—is consistent with the adverse manifestations associated with GC excess such as atrophy and delayed wound healing. Supporting this, aged mice treated with HSD11B1 inhibitors or aged HSD11B1 KO mice featured improvements in skin atrophy and cutaneous healing [105]. 

In a mouse model for metabolic syndrome, and after UV exposure, it was shown that pharmacological blockade of MR reduced oxidative stress ameliorating aging-like skin changes [113]. Importantly, MR was not involved in the long-term UV damage of normal skin suggesting that the pathological aging-like role of MR signaling is specific to metabolic syndrome. Further studies are needed to understand the relative roles of GR and MR in skin aging and to elucidate the mechanisms by which these NRs regulate oxidative stress in physiological and pathological aging.

#### 5.3.2. Cutaneous Inflammation

It has been demonstrated that GR exert key therapeutic roles in experimental mouse models of cutaneous inflammation and cancer due to its anti-proliferative, anti-inflammatory, and anti-tumor functions, mostly mediated by interference with the NF-κB, ERK/AP-1, and signal transducer and activator of transcription 3 (STAT3) signaling pathways (reviewed in References [3,114]). However, MR activation has been clasically assigned to pro-inflammatory and pro-fibrotic actions mainly in the renal and cardiovascular systems [10]. An anti-inflammatory role of GR and MR in adult skin is supported by our findings that adult GR^EKO^ and MR^EKO^ mice showed higher susceptibility to inflammation following exposure to detergent or the phorbol ester PMA, both of which triggered exaggerated proliferative and hyperkeratotic response relative to control mice [13,14]. In response to these stimuli, adult GR^EKO^ and MR^EKO^ skin had up-regulation of ERK and STAT3 activities highlighting the importance of both epidermal NRs in modulating proliferative and inflammatory responses. For GR, these data were further supported by studies in two independently generated mouse models with tamoxifen-induced epidermal deletion of GR in adulthood (K14-Cre-ER^T^//GR^flox/flox^ mice). In these mice, acute PMA treatment triggered increased keratinocyte proliferation and skin inflammation, with pronounced recruitment of polymorphonuclear cells and the expression of TSLP, a key marker of atopic dermatitis, was overinduced and could not be inhibited by GCs [43,68].

Post-translational modification of GR via sumoylation plays a key role in vivo in the susceptibility to skin inflammation. Mice with a mutation in the residue K310 of GR, impairing sumoylation at this site (K293 in humans), showed augmented responses to PMA-induced skin inflammation, which could not be efficiently suppressed by Dex [115,116]. GR K310 sumoylation was required for Dex-induced gene repression through IR nGRE sites as well as interference with NF-κB/AP-1 dependent transcription. The mechanism involves the formation of a repressing complex containing GR, NCoR1/silencing mediator for retinoid or thyroid hormone receptors (SMRT) and HDAC3. In addition, mice with keratinocyte-specific inactivation of NCoR1/SMRT or HDAC3 were also unresponsive to Dex-induced transrepression as tethering of the complex on DNA-bound NF-κB/AP1 was impaired [115,116]. MR post-translational modifications also play an important role though in vivo models are lacking. For instance, MR phosphorylation on S483 lowered its affinity for agonists and inhibited gene transactivation [117]. Regarding transrepression, there is no clear consensus for MR although ChIP studies demonstrated that MR frequently interacts with TF genomic motifs other than MREs via tethering [35].

Despite the pro-inflammatory role for MR in many cell types, the fact that MR^EKO^ mice, similar to GR^EKO^ mice, exhibited worsened responses to skin damage and inflammation with higher cytokine induction relative to controls, indicated that both epidermal corticosteroid receptors act as anti-inflammatory mediators. Moreover, the combined loss of epidermal GR and MR resulted in increased susceptibility, relative to single KOs, to PMA-induced ear edema. This together with the appearance of neutrophil-containing epidermal microabscesses uniquely in DKO mice indicates cooperative anti-inflammatory actions of GR and MR. PMA up-regulated inflammatory markers including *Il6*, *S100a8*, and *Mmp3* in DKO and single KOs relative to controls; with additive increases in Mmp3 expression in DKO relative to single KOs [50]. Importantly, the alterations induced by PMA—including epidermal thickening, increased keratinocyte proliferation, and abnormal K6 expression—were not reduced by Dex treatment in single KO and DKO animals. Finally, DKO were more susceptible than control mice to imiquimod-induced psoriasis with defective epidermal differentiation and microabcesses while single KOs showed an intermediate response. Overall in diseased skin epidermal GR and MR play additive anti-inflammatory roles and both are required for the anti-proliferative and protective actions of GCs.

The importance of local GCs in maintaining skin homeostasis has been recently highlighted by several reports demonstrating that defective de novo GC synthesis in skin contributes to altered skin inflammatory responses in psoriatic patients [60,61,62,63]. It has also been shown that both GR/*NR3C1* and MR/*NR3C2* mRNA levels decrease in psoriatic skin [62,118]. This reduction in GR and MR expression was observed as well in imiquimod-treated WT mouse skin [50], where moreover the expression of GC-target genes was strongly reduced. As expected, GC-target genes showed reduced expression in single KOs and were even more strongly decreased in DKOs. While the DKOs did not develop psoriasis spontaneously, they did show increases in basal Il17f expression, consistent with their higher susceptibility to this disease model.

## 6. Conclusions and Perspectives

Glucocorticoid receptors and MRs are co-expressed in keratinocytes, share high structural and functional homology, can form distinct homo- and heterodimers, theoretically bind to identical regulatory sequences, and interact with common TFs and co-regulators. Altogether, these data strongly suggest that GRs and MRs play common roles in keratinocytes. In fact, in vitro and in vivo studies support this hypothesis as both NRs inhibit epidermal proliferation in healthy skin and counteract inflammation in pathological situations. This poses the question of whether these NRs functionally compensate for each other’s absence in this tissue. Recent data from mice with combined deletion of epidermal GRs and MRs contradicts this hypothesis as the skin phenotype of newborn DKO mice was more severe than that of each individual KO, with additive increases in the up-regulation of several inflammatory markers. How skin alterations resolve in GR^EKO^ or DKO mice remains to be deciphered. Keratinocyte proliferation was similarly increased in single KOs and DKO relative to controls and PMA-induced hyperproliferation and inflammation could not be reduced by treatment with Dex. However, GR and MR played additive anti-proliferative and anti-inflammatory actions in a model of imiquimod-induced psoriasis. On the other hand, it has been demonstrated that in pathological situations MRs mediate some of the adverse effects of GCs such as skin atrophy leading to the question of whether the role of MR is only relevant in pathological situations. As improving GC-based skin therapies is an urgent need, the use of MR antagonists to ameliorate undesired effects related to GC excess is an attractive hypothesis. However, further studies are necessary to clarify the role of MRs in the beneficial or negative side effects of pharmacological GC treatments. Specifically, the identification of the transcriptomic profiles regulated by GRs, MRs, or both, will help determine whether these NRs regulate similar or distinct cellular processes, in particular inflammation. It is also of great interest to unravel the signaling pathways involved in GC-induced transcriptional repression via GR/MR tethering, identifying their respective interactions with other TFs and/or co-regulators. This knowledge could provide a rationale to design selective GC-based strategies that increase anti-inflammatory actions while avoiding impairment of cutaneous barrier function. Finally, the evidence that impaired local GC synthesis in psoriasis lesions has a pathogenic role opens the possibility to restore GC production in psoriatic patients—and potentially in other cutaneous diseases commonly treated with GCs—as novel therapeutic strategy.

## Abbreviations

GCs	Glucocorticoids
MCs	Mineralocorticoids
GR	GC receptor
MR	MC receptor
GRE	GC/MC response element
HSD11B	11 beta hydroxysteroid dehydrogenase
TF	Transcription factor
SC	Stratum corneum
GR^EKO^	GR epidermal KO
MR^EKO^	MR epidermal KO
DKO	Double GR/MR epidermal KO
HDAC	Histone deacetylase
HSP	Heat shock protein
NF-κB	Nuclear factor-kappaB
AP-1	Activator protein 1
MAPK	Mitogen-activated protein kinase
ERK	Extracellular signal regulated kinase
SMRT	Silencing mediator for retinoid or thyroid hormone receptors
